# Enhancing the Fire Safety and Smoke Safety of Bio–Based Rigid Polyurethane Foam via Inserting a Reactive Flame Retardant Containing P@N and Blending Silica Aerogel Powder

**DOI:** 10.3390/polym13132140

**Published:** 2021-06-29

**Authors:** Guangxu Bo, Xiaoling Xu, Xiaoke Tian, Jiao Wu, Yunjun Yan

**Affiliations:** Key Laboratory of Molecular Biophysics of the Ministry of Education, College of Life Science and Technology, Huazhong University of Science and Technology, Wuhan 430074, China; B13991359@163.com (G.B.); xuxiaoling@hust.edu.cn (X.X.); 18237396357@163.com (X.T.); 18390233073@163.com (J.W.)

**Keywords:** tetraethyl(1,5–bis(bis(2–hydroxypropyl)amino)pentane–1,5–diyl)bis(phosphonate), SA powder, castor oil–based rigid polyurethane foam, fire and smoke safety, building insulation materials

## Abstract

Rigid polyurethane foams (RPUFs) are widely used in many fields, but they are easy to burn and produce a lot of smoke, which seriously endangers the safety of people’s lives and property. In this study, tetraethyl(1,5–bis(bis(2–hydroxypropyl)amino)pentane–1,5–diyl)bis(phosphonate) (TBPBP), as a phosphorus–nitrogen–containing reactive–type flame retardant, was successfully synthesized and employed to enhance the flame retardancy of RPUFs, and silica aerogel (SA) powder was utilized to reduce harmful fumes. Castor oil–based rigid polyurethane foam containing SA powder and TBPBP was named RPUF–T45@SA20. Compared with neat RPUF, the obtained RPUF–T45@SA20 greatly improved with the compressive strength properties and the LOI value increased by 93.64% and 44.27%, respectively, and reached the V–0 rank of UL–94 testing. The total heat release (THR) and total smoke production (TSP) of RPUF–T45@SA20 were, respectively, reduced by 44.66% and 51.89% compared to those of the neat RPUF. A possible flame–retardant mechanism of RPUF–T45@SA20 was also proposed. This study suggested that RPUF incorporated with TBPBP and SA powder is a prosperous potential composite for fire and smoke safety as a building insulation material.

## 1. Introduction

Rigid polyurethane foams (RPUFs) are widely used in many fields, especially in the construction field as insulation materials, due to high compressive strength, low thermal conductivity, convenient manufacture and cost effectiveness [1,2,3,4]. Neat RPUF is flammable and diffuses rapidly during combustion as a result of the low break–up temperature and high pore structure with extensive multi–contact between matrix and air [5,6]. Therefore, in order to make RPUF play a better role in the field of building insulation, it is particularly important to endow RPUF with good flame–retardant properties. Research on flame–retardant RPUFs at home and abroad was found in many published articles [7,8,9,10,11,12,13,14].

Reactive flame retardants have been proved to enhance the flame retardancy of RPUFs; however, RPUFs inserting reactive flame retardants produce more smoke than neat RPUF when they burn. Therefore, the fire safety and smoke safety of RPUFs can be improved by using flame retardants and smoke suppressors at the same time. In our previous study [15], silica aerogel (SA) powder was proved to remarkably enhance compressive strength and reduce harmful fumes.

Hence, a phosphorus–nitrogen–containing reactive flame retardant, tetraethyl (1,5–bis(bis(2–hydroxypropyl)amino)pentane–1,5–diyl)bis(phosphonate) (TBPBP), was firstly synthesized and employed to improve the flame–retardant property of castor oil–based RPUFs, and SA powder was used with TBPBP for enhancing properties and reducing smoke. Taking into account the practicality of foam preparation, the types of foam catalysts and the addition amounts of SA powder are, respectively, different in this manuscript and the article [15]. The flame–retardant preference and physical properties of castor oil–based RPUFs were characterized via various test equipment. Meanwhile, a possible flame–retardant mechanism of RPUF incorporated with TBPBP and SA powder was also proposed.

## 2. Materials and Methods

### 2.1. Raw Materials

Modified castor oil polyols (MCOP) (hydroxyl value = 296 mgKOH/g, viscosity = 575 mPa·s) were purchased from Shanghai Joule New Material Tech. Co., Ltd., Shanghai, China. Glutaric dialdehyde (25%, *g*/*g*), tris(dimethylaminomethyl)phenol (DMP–30), polyethylene glycol–200 (PEG–200), diisopropanolamine, and diethyl phosphite (all C.P. grade) were bought from China National Pharmaceutical Group Co., Ltd., Beijing, China. Polymeric diphenylmethane diisocyanate (pMDI) 44v20 (NCO% = 30.0–32.0) and silicone oil (all T.P. grade) were commercially obtained from Chengdu Advanced Polymer Tech. Co., Ltd., Chengdu, China. Deionized water and silica aerogel powder were generated in our laboratory.

### 2.2. Preparation of Tetraethyl (1,5–bis(bis(2–hydroxypropyl)amino)pentane–1,5 –diyl)bis(phosphonate) (TBPBP)

**Scheme 1:**
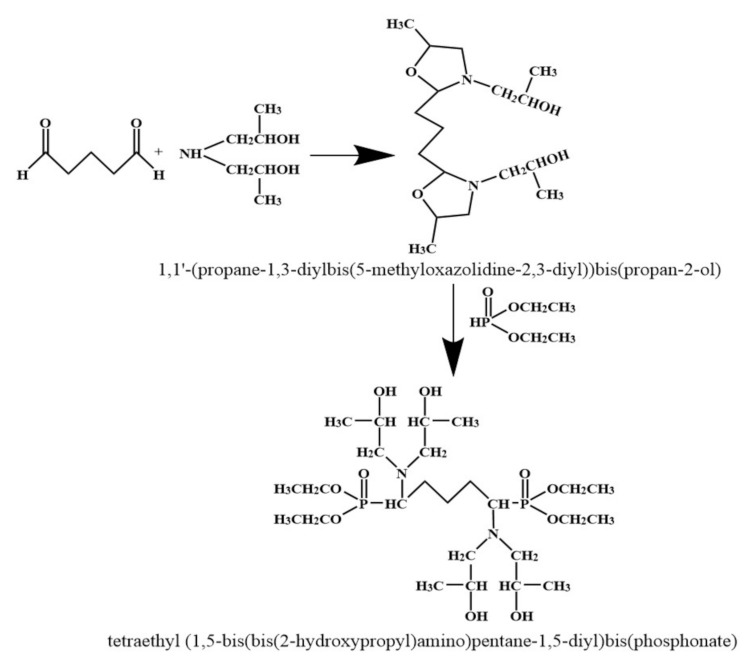
The synthetic route of TBPBP.

**Figure 1:**
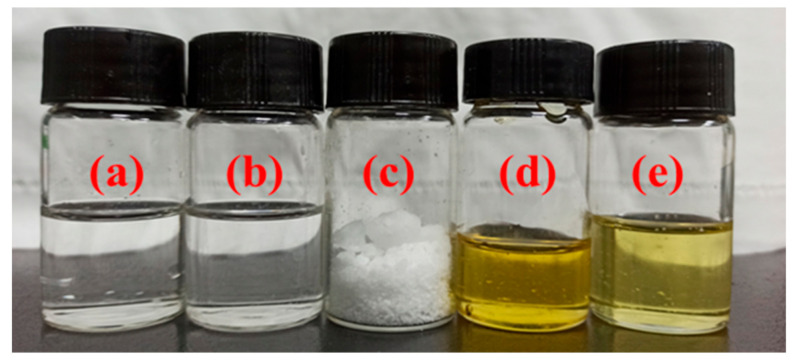
A photo of raw materials and TBPBP: (**a**) glutaric dialdehyde, (**b**) diethyl phosphite, (**c**) diisopropanolamine, (**d**) intermediate, (**e**) TBPBP.

Diisopropanolamine was put into a 500 mL single–mouth glass flask, and then was placed in warm oil bath at 60 °C for obtaining liquid diisopropanolamine. Glutaric dialdehyde (the molar ratio of glutaric dialdehyde to diisopropanolamine was 1:2) was added into the flask, which was equipped with condensing pipe and placed in warm oil bath at 60 °C, then mixed reagent was stirred at 400 rpm via a magnet for 3 h. After this, the yellow intermediate in the flask was obtained by rotating evaporation. Afterwards, diethyl phosphite (the molar ratio of glutaric dialdehyde to diethyl phosphite was 1:2) was slowly dropped into the flask, which was placed in warm oil bath at 75 °C, and mixed reagent was stirred at 400 rpm via a magnet for 3 h. Finally, yellowish TBPBP was gained by rotating evaporation. The synthetic route of TBPBP is presented in Scheme 1. Figure 1 is a photo of raw materials and TBPBP.

### 2.3. Preparation of Castor Oil–Based Rigid Polyurethane Foams

Modified castor oil polyols, polyethylene glycol–200, tetraethyl (1,5–bis(bis(2–hydroxypropyl)amino)pentane–1,5–diyl)bis(phosphonate) and polymeric diphenylmethane diisocyanate were used as basic materials. Deionized water was a blowing agent. Tris(dimethylaminomethyl)phenol was a catalyst. Silicone oil acted as a foam stabilizer.

The various reagents listed in Table 1, except for polymeric diphenylmethane diisocyanate, were added into a plastic beaker, and stirred at 400 rpm for 10 min. Then, the weighed polymeric diphenylmethane diisocyanate was poured into the beaker at 1100 rpm. After ca. 10 s, the mixed components were poured into a steel plate at 60 °C for free foaming. After about 3 min, the steel plate was placed in a vacuum drying oven and dried at 60 °C for 10 h.

**Scheme 2:**
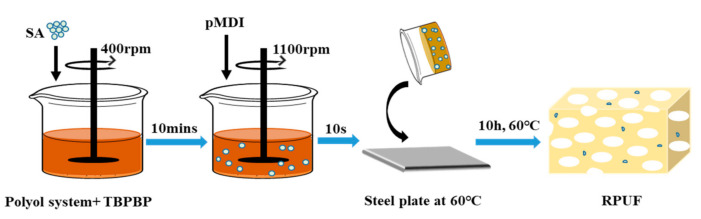
Schematic diagram of preparation of RPUF–T45@SA20.

The reaction formula for preparing RPUFs is given in Scheme S1. Schematic diagram of preparation of RPUF–T45@SA20 is shown in Scheme 2. The preparation processes of other RPUFs were similar to that of RPUF–T45@SA20.

### 2.4. Characterization

Liquid Chromatography–Mass Spectrometry (LC/MS) curve of TBPBP was gained from an Orbitrap LC/MS (Q Exactive) (Thermo Fisher Scientific Technology Co., Ltd., Waltham, Massachusetts, USA).

Fourier–transform infrared (FT–IR) spectra with the wave number ranging from 400 to 4000 cm^−1^ were performed by a Nicolet iS50R spectrometer (Thermo Fisher Scientific Technology Co., Ltd., Waltham, Massachusetts, USA) using KBr slices.

NMR spectra of TBPBP were attained on an Ascend TM 600 MHZ NMR apparatus (Bruker company, Karlsruhe, Germany). DMSO–d6 was selected as NMR reagent.

X–ray photoelectron spectroscopy (XPS) curves were recorded by an AXIS SUPRA+ spectrometer (Shimadzu Kratos Corporation, Kyoto, Japan) using Al Ka excitation radiation (hv–1200 eV).

The thermal conductivity of RPUFs (30 × 30 × 30 mm^3^) was determined by a TPS2500S (Hot Disk, Uppsala, Sweden) according to ISO 22007–2:2008.

FSEM (EM–30 Plus, COXEM Co., Ltd., Daejeon City, South Korea) was used to observe the section morphology and carbon residue morphology of RPUF. The sample was uniformly coated with a thin layer of platinum before testing, and the relative elemental composition of the surface was determined by an energy dispersive X–ray (EDSX) spectrometer.

Thermal gravimetric analysis (TGA) was tested by a STA2500 thermal analyzer (STA2500, NETZSCH Corporation, Selb, Bavaria, Germany). Samples of 3–10 mg were heated from 30 °C to 700 °C with a heating rate at 10 °C/min under nitrogen air atmosphere, respectively.

The combustion behavior of RPUF was analyzed using a cone calorimeter (FTT I–Cone 0402, Europe and America Dadi Technology Group, Hong Kong, China) according to ASTM E1354–17. The sample size was 100 × 100 × 30 mm^3^ and the external heat flux was 35 kW.

The pyrolysator (PY 2020, Shimadzu Kratos Corporation, Kyoto, Japan) and the combined GCMS–QP2010 SE system were used for the pyrolysis gas chromatography–mass spectrometry (PY–GC/MS) with helium as the protective gas. The sample (about 300 mg) was heated from 30 °C to 650 °C at a rate of 1000 °C/min and held for 20 s before flowing into the column. The MS indicator was in electron shock mode (electron energy was 70 eV) and the ion source temperature was maintained at 180 °C.

The tests of density, compressive strength, limiting oxygen index, vertical burning tests, and laser Raman spectroscopy of RPUFs refer to the article [15].

## 3. Results and Discussion

### 3.1. Characterization of TBPBP

**Figure 2:**
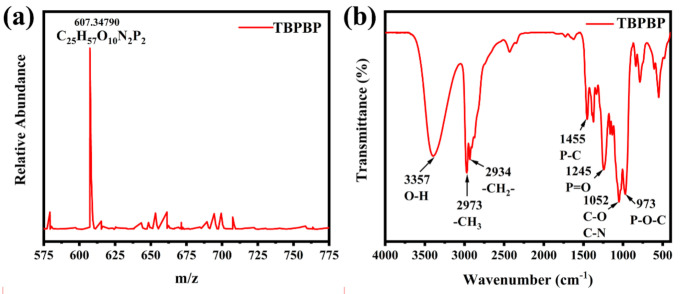
The LC/MS curve (**a**) and FT–IR spectrum (**b**) of TBPBP.

TBPBP was a vital reagent containing hydroxyl groups, which could react with pMDI. Meanwhile, it was also a flame retardant because of the existence of flame–retardant P and N elements. Therefore, the properties of TBPBP undoubtedly affected the properties of RPUFs, including physical properties and flame–retardant properties. The chemical structure and physical property of the fabricated TBPBP were characterized by LC/MS, FT–IR, NMR spectra and a thermal analyzer. The LC/MS curve of TBPBP is displayed in Figure 2a. *m*/*z* = 607.34790 and C_25_H_57_O_10_N_2_P_2_ mean the successful synthesis of TBPBP, although its actual molecular weight and molecular formula were 606.7 and C_25_H_56_O_10_N_2_P_2_, respectively. Figure 2b shows the FT–IR spectrum of TBPBP. Vital peaks at 3357 cm^−1^, 2973 cm^−1^, 2934 cm^−1^, 1455 cm^−1^, 1245 cm^−1^, 1052 cm^−1^, and 973 cm^−1^ were present, which were ascribed to the stretching vibrations of O–H, –CH_3_, –CH_2_–, P–C, P=O, C–O and C–N, P–O–C, respectively. Actually, those groups all existed in TBPBP.

**Figure 3:**
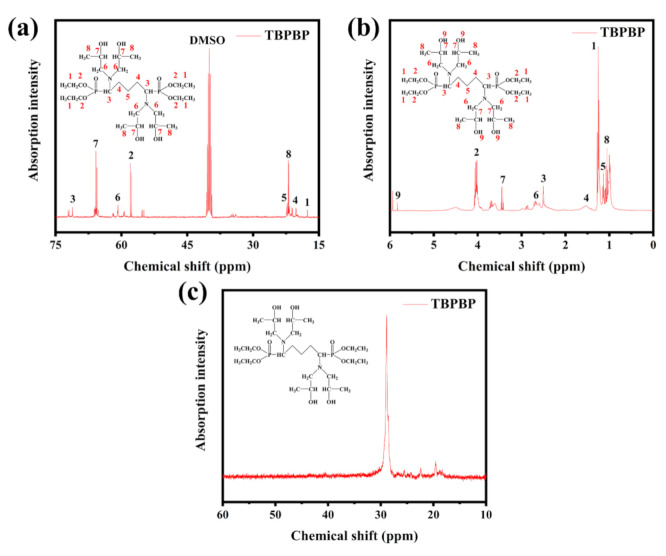
NMR spectra of TBPBP: ^13^C NMR (**a**), ^1^H NMR (**b**), ^31^P NMR (**c**).

**Figure 4:**
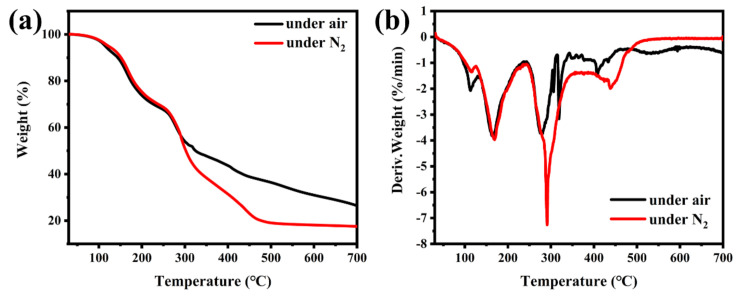
TGA curves (**a**) and DTG curves (**b**) of TBPBP under N_2_ and air.

Figure 3 displays the ^13^C NMR, ^1^H NMR and ^31^P NMR spectra of TBPBP, and the peaks are mainly in accord with the predicted peaks of TBPBP. For example, the ^31^P NMR spectrum of TBPBP in Figure 3c shows a single signal at 28.84 ppm. TGA and DTG curves of TBPBP under N_2_ and air are shown in Figure 4. It is worth noting that the residue weight at 700 °C under air of TBPBP was 26.46%, which was higher than 17.55% under N_2_. As the intermediate products of the decomposition of TBPBP under air reacted with oxygen, this introduced O element into the residue [16].

The characterization via LC/MS, FT–IR, NMR spectra and the thermal analyzer revealed that TBPBP was successfully synthesized, and it was suitable to act as a reactive–type flame retardant for enhancing the physical properties and flame–retardant properties of RPUFs.

### 3.2. Characterization of RPUFs

#### 3.2.1. Chemical Component

**Figure 5:**
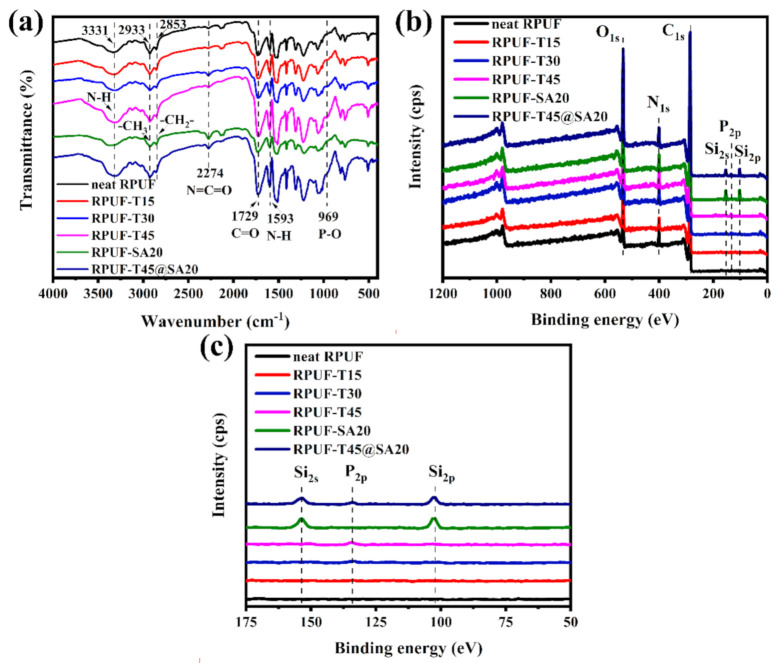
FT–IR spectra (**a**) and XPS curves (**b**), (**c**) of RPUFs.

The chemical structures of RPUFs were characterized via FT–IR and XPS. FT–IR spectra are shown in Figure 5a. Distinct absorption peaks of all RPUFs at 3331 cm^−1^, 2933 cm^−1^, 2853 cm^−1^ and 1729 cm^−1^ were attributed to the stretching vibrations of N–H, –CH_3_, –CH_2_– and C=O, respectively. Additionally, 2274 cm^−1^ for N=C=O anti–symmetric stretching vibrations and 1593 cm^−1^ for N–H bending vibrations existed in the FT–IR spectrum of all RPUFs. These peaks all consisted with articles [17,18]. Notably, 969 cm^−1^ for P–O stretching vibrations was found in the FT–IR spectrum of RPUF–T15, RPUF–T30, RPUF–T45 and RPUF–T45@SA20, which revealed that TBPBP was introduced into the RPUF matrix. However, absorption peaks of SA powder were not observed in RPUF–SA20 and RPUF–T45@SA20, which did not mean RPUF–SA20 and RPUF–T45@SA20 had no SA powder. To further examine the chemical structure of RPUFs, an X–ray photoelectron spectrometer was employed for obtaining XPS curves. Figure 5b, c present XPS curves of RPUFs. The curve of neat RPUF only finds C_1s_, N_1s_, and O_1s_. However, the curves of RPUF–T15, RPUF–T30 and RPUF–T45 show P_2p_ besides C_1s_, N_1s_, and O_1s_, and the peaks of P_2p_ became surely obvious from RPUF–T15 to RPUF–T45, which resulted from the increasing additive amount of TBPBP. In the P_2p_ spectra, the peak at 133.7 eV was assigned to the P–O–C group [19]. Si2s and Si2p were found in the curve of RPUF–SA20 due to the addition of SA powder. RPUF–T45@SA20 contained TBPBP and SA powder, so its curve showed five elements, namely C, N, O, Si, and P. The results of FT–IR spectra and XPS curves together proved that all RPUFs were successfully prepared.

#### 3.2.2. Surface Microstructure, Mapping Energy Spectrum Analyses

**Figure 6:**
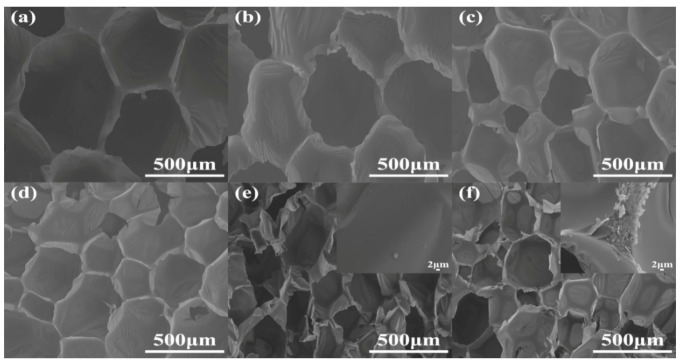
SEM images of neat RPUF (**a**), RPUF–T15 to RPUF–T45@SA20 (**b**–**f**).

The cell morphology is a vital, important factor that affects the physical and mechanical properties of RPUFs [20]. The cross sections of RPUFs were characterized by SEM and EDXS, and the corresponding SEM images are presented in Figure 6. Obviously, by the insertion of increasing TBPBP into the copolyester chains of RPUFs, the cell number and size became larger and smaller, respectively. This was because TBPBP had an emulsifying effect. Compared to neat RPUF, RPUF–SA20 and RPUF–T45@SA20 possessed a larger cell number and smaller cell size. Meanwhile, the utilization of SA powder caused rougher cross sections than neat RPUF, RPUF–T15 to RPUF–T45. The rougher cross sections possibly caused by cutting, before the samples of RPUF–SA20 and RPUF–T45@SA20, were characterized via SEM. Interestingly, a single SA particle was found on the surface of RPUF–SA20, while a cluster of SA particles was observed at the junction of cells. The phenomenon was caused by the different viscosities of polyol systems that fabricated RPUF–SA20 and RPUF–T45@SA20. EDXS images of RPUFs are shown in Figure S1. The results of EDXS were consistent with the results of XPS.

#### 3.2.3. Physical and Mechanical Properties

The application of RPUFs as thermal insulation materials is affected by physical and mechanical properties, especially density, compressive strength, and the thermal conductivity coefficient. The density, compressive strength and thermal conductivity of RPUFs are all listed in Table 2. In terms of the density of foams, this depends on the amount of the base raw material used to form a network [21]. The density of RPUFs gradually increased with the increase in TBPBP or SA powder contents. Two reasons were responsible for the increasing density. The first one was that TBPBP or SA powder had a higher density than the neat RPUF matrix. The other was that TBPBP or SA powder increased the viscosity of the polyol system, which partly impeded the foaming process.

From Table 2, the compressive strength of RPUFs containing TBPBP or SA powder was larger than neat RPUF. The compressive strength of neat RPUF was 236 ± 29 kPa, while the compressive strengths of RPUF–T45, RPUF–SA20, and RPUF–T45@SA20 were, respectively, increased to 422 ± 30 kPa, 396 ± 18 kPa and 457 ± 24 kPa, and correspondingly obtained improved compressive strength properties with an increment of 78.81%, 67.79% and 93.64%. Generally, the compressive strength of RPUFs mainly relies on density, the rigidity of the polymer matrix, and the cellular size and number [22]. Larger density is apt to cause higher compressive strength. Undoubtedly, TBPBP and SA were used together to gain the highest compression performance for RPUF.

Like density and compressive strength, thermal conductivity is also an important parameter for RPUFs as thermal insulation materials. As the contents of TBPBP and SA powder, RPUFs obtained higher thermal conductivity than neat RPUF. Three reasons might be attributed to the result. First, TBPBP and SA powder had higher thermal conductivity than the neat RPUF matrix. Second, the increase in density increased the thermal conductivity for RPUFs due to the progressively accrescent contribution of solid thermal conductivity [23]. Third, the use of TBPBP and SA powders altered the foaming process, affecting the opening ratio, cell wall thickness, cell size, and cell wall conduction and ventilation conduction along the cell wall. The air is higher than for the CO_2_ (0.025 vs. 0.015 mW/(mK)) [24,25]. Cell size and cell wall thickness influence photon transfers and thus affect the thermal conductivity of RPUFs [26].

In summary, the use of TBPBP and SA significantly improved compression strength and slightly worsened density and thermal conductivity for RPUFs.

#### 3.2.4. Thermal Stability

Consistent with our understanding, good thermal stability can improve the service life of RPUFs at high temperatures. Thermal gravimetric analysis is an important analysis for evaluating the thermal properties of RPUFs [27]. The thermal stability of RPUFs under N2 and air was tested via a thermal analyzer.

**Figure 7:**
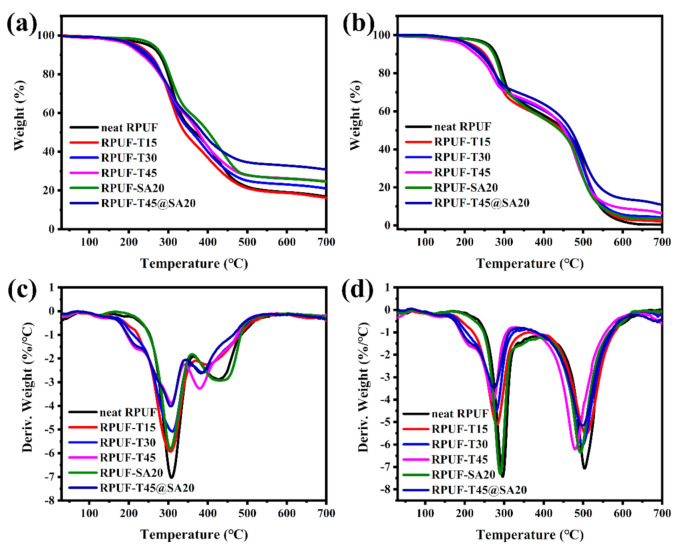
TGA curves of RPUFs under N_2_ (**a**) and under air (**b**), DTG curves of RPUFs under N_2_ (**c**) and under air (**d**).

The curves of TGA and DTG are exhibited in Figure 7. From Figure 7, not only under N_2_ but also under air, there are two main degradation processes for all RPUFs, which is consistent with previous reports [8,28]. In terms of two degradation processes of all RPUFs, the first was attributed to the degradation of hard segments, and the second was ascribed to the degradation of soft segments. The data of the 5% weight loss temperature (T_5%_), the 50% weight loss temperature (T_50%_), the maximum rate of weight loss (R_max_), the temperature of maximum rate (T_max_), and the residue yield are listed in Table 3 (under N_2_) and Table 4 (under air).

With the increasing incorporation of TBPBP into the RPUF matrix, T_5%_ came earlier and earlier under N_2_ and air, which was caused by the degradation of TBPBP, as mentioned above, while T_50%_ did not conform to such a rule. T_max1_ of RPUFs was similar under N_2_ and air; however, R_max1_ became lower with increasing amounts of TBPBP. Low R_max_ means a low peak heat and smoke release, which is beneficial to fire safety. As the contents of TBPBP increased, R_max2_ did not decrease significantly under N_2_ and air. The residue yield at 700 °C was greatly improved with introducing TBPBP and SA powder into RPUFs under both nitrogen and air. Specifically speaking, the residue yield of neat RPUF was, respectively, 16.88% and 0.47% under N_2_ and air, while the residue of RPUF–T45@SA20 reached up to 30.91% and 10.96%. The results of TGA and DTG revealed TBPBP or SA powder improved thermal stability for the foams, especially both the utilization of TBPBP and SA powder.

#### 3.2.5. Flame Behavior

LOI testing and UL–94 testing are both important tests to assess the flame retardancy of polymers [29]. The LOI value indicates the lowest oxygen content to maintain combustion [9]. High LOI values and UL–94 grades disclose good flame–retardant performance for RPUFs [30]. LOI values and UL–94 grades are summarized in Table 5. From Table 5, the LOI values of RPUF–T15, RPUF–T30, RPUF–T45, RPUF–SA20, and RPUF–T45@SA20 were, respectively, 22.9, 23.4, 25.6, 20.3, and 27.7, but the LOI value of neat RPUF was only 19.2. TBPBP greatly improved the flame retardancy of RPUFs, while SA powder failed to enhance the flame retardancy. However, the simultaneous use of TBPBP and SA significantly improved the flame–retardant performance for RPUF–T45@SA20, which means that TBPBP and SA powder generated a synergistic effect on enhancing the flame–retardant performance. In terms of UL–94 tests, neat RPUF and RPUF–SA20 both gained N.R., while RPUF–T15, RPUF–T30, RPUF–T45, and RPUF–T45@SA20 all achieved V–0 rank owing to the formation of dense char residues that TBPBP facilitated to create.

#### 3.2.6. Surface Microstructure, Mapping Energy Spectrum Analyses of Char Residues

**Figure 8:**
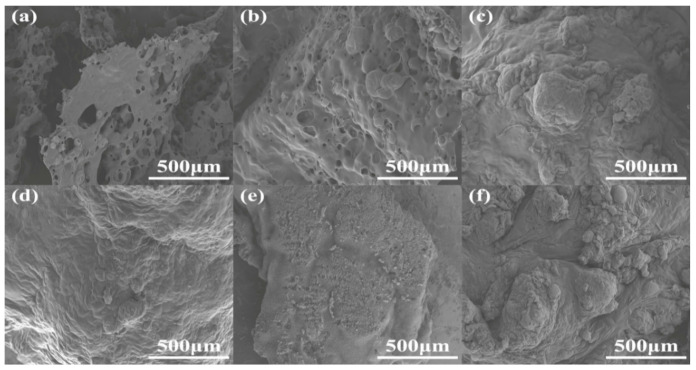
SEM images of char residues of neat RPUF (**a**), RPUF–T15 to RPUF–T45@SA20 (**b**–**f**).

The char residues of RPUFs after burning via ca. 1300 °C flame were characterized via SEM and EDXS, and the corresponding SEM images are presented in Figure 8. From Figure 8, the char residue of neat RPUF was loose, holey and brittle, which could not protect the interior matrix to avoid combustion. This was the reason that neat RPUF gained a low LOI value and UL–94 grade. As the contents of TBPBP increased, the char residues were intumescent and became more intact and denser, which could protect the interior matrix to avoid the exchange with heat and oxygen and played the role of flame retardant performance. Although the char residue of RPUF–SA20 was dense and intact, it was formed at the late combustion phase. The reason for the formation of dense and intact residue was that the external migration of SA powder enhanced the viscosity of the residue in the late combustion phase, which could not enhance the fire safety. However, the simultaneous use of TBPBP and SA facilitated the formation of dense and intact residue, which made RPUF–T45@SA20 effectively undergo a big fire. The corresponding EDXS images of the char residues of RPUFs are shown in Figure S2. EDXS images of the char residue of RPUF–T45@SA20 show P and Si, except for C, N, and O elements. The result showed that P and Si elements were vital flame–retardant elements, and TBPBP acted as a flame retardant that played the role of the condensed phase.

#### 3.2.7. Raman Tests for Char Residues

**Figure 9:**
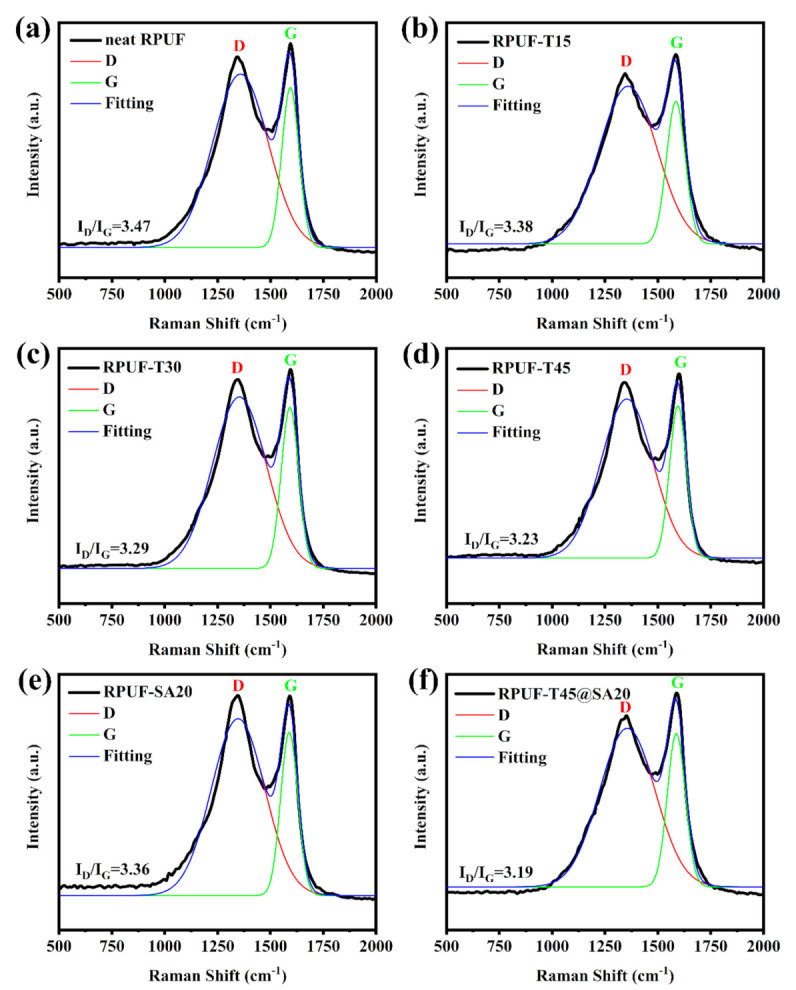
Raman curves of char residues of neat RPUF (**a**), RPUF–T15 to RPUF–T45@SA20 (**b**–**f**).

Generally, Raman spectroscopy is considered to characterize the graphitization degree of post–combustion carbonaceous materials including RPUFs, epoxy resins, and so forth [9]. The graphitization degree of char residues is calculated by the D and G band intensity ratio [31]. The intensity of the D band is assigned to amorphous carbon, and the intensity of the G band is ascribed to graphitized carbon [12]. A lower graphitization degree means more stable char residues. Figure 9 shows Raman curves of char residues of all RPUFs. The graphitization degree was 3.47, and the graphitization degree of RPUF–T15, RPUF–T30, RPUF–T45, RPUF–SA20, and RPUF–T45@SA20 was decreased to 3.38, 3.29, 3.23, 3.36, and 3.19, respectively. Obviously, the utilization of TBPBP was beneficial for obtaining stable char residues for RPUFs. Meanwhile, the simultaneous use of TBPBP and SA powder obtained the most stable char residue in comparison with the rest of the RPUFs due to their synergistic effect. Stable char residue was responsible for the high LOI value and V–0 rank of UL–94 testing for RPUF–T45@SA20.

#### 3.2.8. Cone Calorimeter Testing

**Figure 10:**
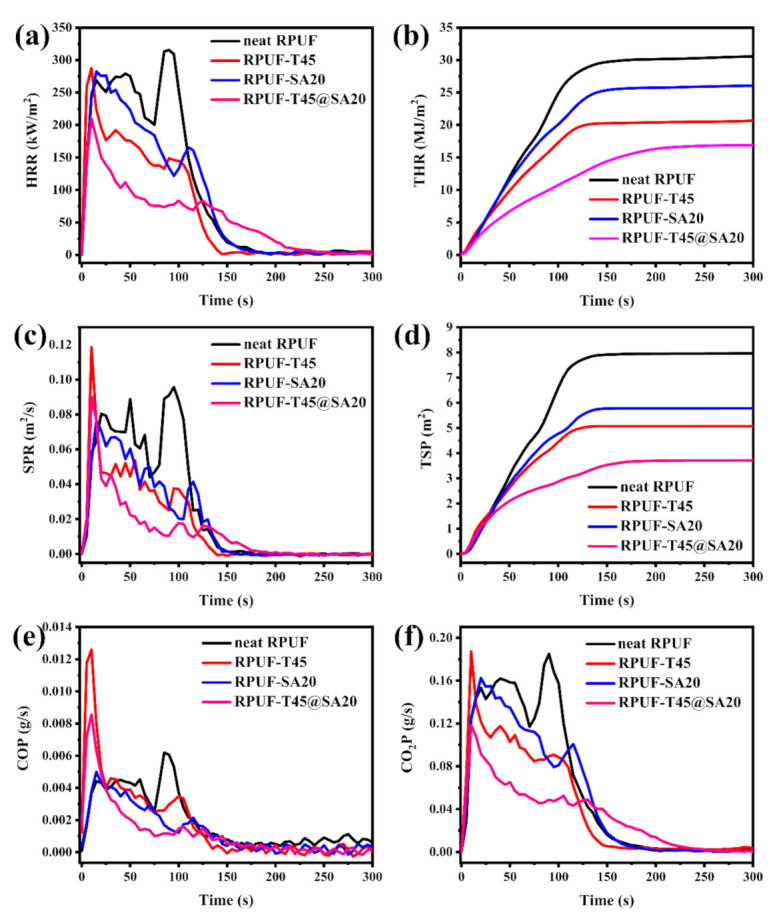
HRR curves (**a**), THR curves (**b**), SPR curves (**c**), TSP curves (**d**), COP curves (**e**) and CO_2_P curves (**f**) of neat RPUF, RPUF–T45, RPUF–SA20 and RPUF–T45@SA20.

Cone calorimeter (CC) testing is a widely used vital test with regard to simulating real combustion for RPUFs, which belongs to the big fire test as opposed to the small fire test of LOI and UL–94. According to the above results, neat RPUF, RPUF–T45, RPUF–SA20 and RPUF–T45@SA20 were selected to carry out CC testing. Some important indicators including the heat release rate (HRR), total heat release rate (THR), smoke production rate (SPR), total smoke production (TSP), CO production (COP) and CO_2_ production (CO_2_P) are presented in Figure 10.

As shown in Figure 10a, HRR curves have more than one peak, which is due to the combustion process of intermediary decomposition products, intermediary char or even due to the release and combustion of volatiles [32,33]. In rough order of the peak of the heat release rate (pHRR) was neat RPUF, RPUF–SA20, RPUF–T45, and RPUF–T45@SA20. In particular, RPUF–T45@SA20 had the lowest pHRR compared to other foams, which means that the synchronous introduction of TBPBP and SA powder into RPUF effectively reduced pHRR. Low pHRR is favorable for fire safety. From Figure 10b, THR curves of neat RPUF, RPUF–T45, RPUF–SA20 and RPUF–T45@SA20 are, respectively, 30.54 MJ/m^2^, 20.67 MJ/m^2^, 26.06 MJ/m^2^, and 16.90 MJ/m^2^. Following RPUFs’ formula, the utilization of TBPBP was facile to obtain low THR in comparison with SA powder. Meanwhile, the concurrent use of TBPBP and SA powder was beneficial to gain the lowest THR, which was in complete agreement with HRR results.

As presented in Figure 10c, SPR curves are broadly similar to HRR curves. Nevertheless, the peak of the smoke production rate (pSPR) was a little bit different from pHRR. The first pSPR of RPUF–T45 and RPUF–T45@SA20 was, respectively, higher than those of neat RPUF and RPUF–SA20 because of the decomposition of TBPBP. In addition, the pSPR of RPUF–SA20 was lower than that of neat RPUF due to the three–dimensional spatial structure of SA [15]. From Figure 10d, TSP curves of neat RPUF, RPUF–T45, RPUF–SA20 and RPUF–T45@SA20 are, respectively, 7.69 m^2^, 5.07 m^2^, 5.78 m^2^ and 3.70 m^2^. Evidently, TBPBP and SA powder were both conducive to reducing TSP for RPUF. Moreover, the coinstantaneous utilization of TBPBP and SA powder made the TSP of RPUF–T45@SA20 decrease by 51.89% compared to that of neat RPUF. RPUF–T45@SA20 with a low TSP means better smoke safety than other RPUFs.

We all know that CO and CO_2_ cannot support breathing for humans and animals. In particular, CO binds to hemoglobin in the blood, which is toxic and even life–threatening for humans. Unfortunately, the introduction of TBPBP leads to the max release rate of CO between 5 s and 25 s after RPUF ignition, as shown in Figure 10e. As TBPBP promoted the formation of dense char residues, this affected the complete combustion of the internal matrix. In order to overcome this weakness, SA powder with a three–dimensional spatial structure was simultaneously used with TBPBP to fabricate RPUF–T45@SA20. Compared to RPUF–T45, the max release of CO of RPUF–T45@SA20 was decayed ca. 30%. In addition, the release amount of CO_2_ of RPUF–T45@SA20 was the lowest among the foams.

**Figure 11:**
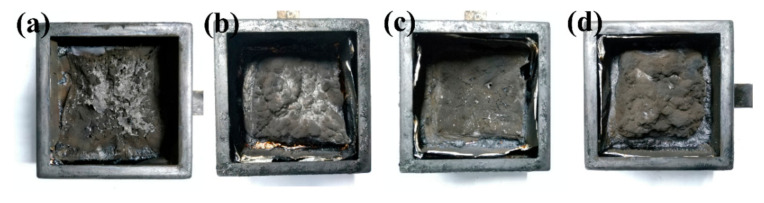
The digital photos of char residues after cone calorimeter testing of neat RPUF (**a**), RPUF–T45 (**b**), RPUF–SA20 (**c**) and RPUF–T45@SA20 (**d**).

Some crucial parameters, such as the time to ignition (TTI), THR, TSP, average effective heat combustion (av–EHC) and char residue, are listed in Table 6. The TTI of neat RPUF and RPUF–SA20 was 0 s, while the TTI of RPUF–T45 and RPUF–T45@SA20 was 3 s and 4 s, respectively. Large values of TTI mean that RPUFs are hard to ignite and possess prominent flame–retardant performance. The value of av–EHC of neat RPUF was 21.36 MJ/kg, and the values of RPUF–T45, RPUF–SA20, and RPUF–T45@SA20 were 15.75 MJ/kg, 19.69 MJ/kg, and 13.04 MJ/kg, respectively. A low value of av–EHC displays the incomplete combustion of the volatile gases in the gas phase for RPUFs [34]. The lowest value of av–EHC of RPUF–T45@SA20 among RPUFs resulted from the firm, intact, intumescent and holistic char layer via the synergistic effect of TBPBP and SA powder. The photos of char residues of the CC testing of RPUFs are presented in Figure 11. From Figure 11, the firm, intumescent and holistic char layers of RPUF–T45 and RPUF–T45@SA20 are found, which are consistent with the high residue yield of Table 6.

The results of cone calorimeter testing, indicating the synergistic effect of TBPBP and SA powder in enhancing fire and smoke safety, were found for RPUF–T45@SA20.

#### 3.2.9. Py–GC/MS Analysis of RPUF–T45 and FI–IR Analysis of Its Residue

**Figure 12:**
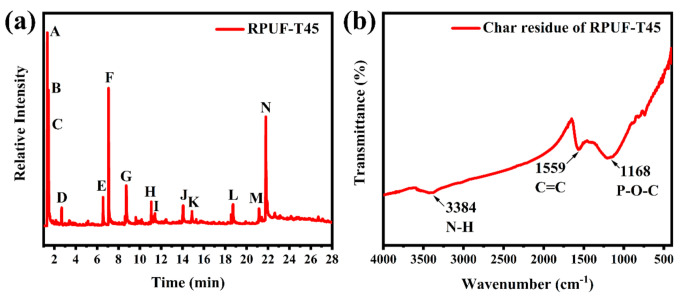
Pyrogram of RPUF–T45 at 650 °C (**a**) and FT–IR spectrum of char residue of RPUF–T45 (**b**).

Pyrolysis gas chromatography and mass spectrometry (Py–GC/MS) is a method to study the flame–retardant mechanism in the gaseous phase in–depth by analyzing the pyrolytic products of samples [35]. The pyrolysis behavior of RPUF–T45 (Figure 12a) was performed via Py–GC/MS at a temperature of 650 °C to further reveal the flame–retardant performance of TBPBP. Chemical structures of the main peaks are listed in Table S1. Peak A at 1.34 min was designated CO_2_, and peak E at 6.55 min and peak F at 7.07 min were attributed to diethyl phosphite and aniline, respectively. Peak N was ascribed to 4,4′–diaminodiphenylmethane. Diethyl phosphite was derived from the decomposition of TBPBP, while aniline and 4,4′–diaminodiphenylmethane were dated from the hard segment degradation of RPUF. The char residue of RPUF–T45 was tested via FT–IR, and the corresponding spectrum is shown in Figure 12b. From Figure 12b, the absorption peaks at 3384 cm^−1^, 1559 cm^−1^ and 1168 cm^−1^ were due to the stretching vibrations of N–H, C=C and P–O–C, respectively. The discovery of C, N, O, and P elements via FT–IR was consistent with EDXS images of the char residue of RPUF–T45.

The results of the pyrogram of RPUF–T45 and the FT–IR spectrum of the char residue of RPUF–T45 demonstrated that TBPBP acted as a flame retardant and played a role in both condensed and gas phases during pyrolysis.

### 3.3. Possible Flame–Retardant Mechanism

**Figure 13:**
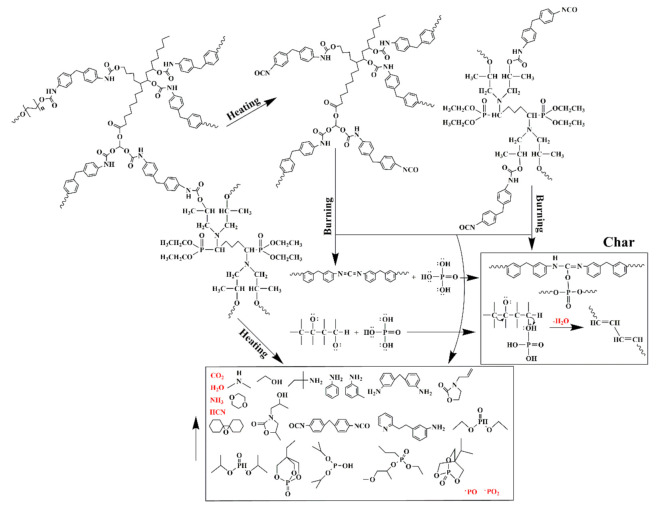
A possible flame–retardant mechanism for RPUFs inserted with TBPBP.

**Scheme 3:**
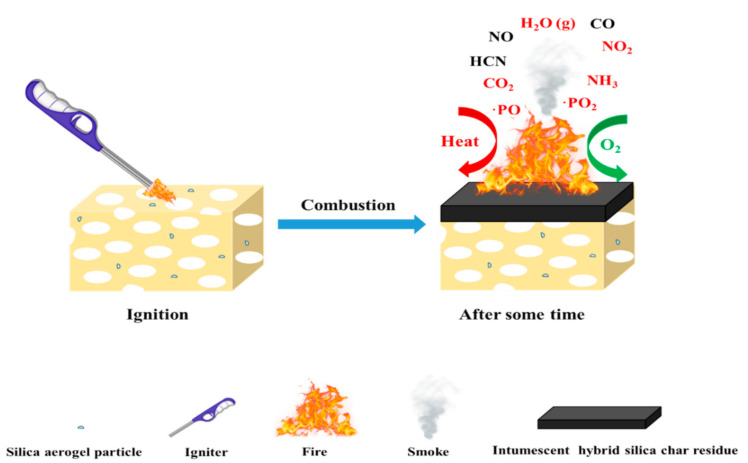
A possible flame–retardant mechanism for RPUF–T45@SA20.

Based on the above characterization results, a possible flame–retardant mechanism of RPUFs inserted with TBPBP is shown in Figure 13. The decomposed products of RPUFs inserted with TBPBP mainly included CO_2_, NH_3_, H_2_O, diethyl phosphite, aniline, and 4,4′–diaminodiphenylmethane. The decomposed products primarily played a flame retardant role in the gas phase via diluting the combustible molecules and simultaneously going away with some heat. Phosphorus–containing products and nitrogen–containing compounds combined with carbon to form a phosphorous–nitrogen–containing carbonaceous structure in the condensed phase [16]. Therefore, TBPBP definitely played a flame–retardant role in both condensed and gas phases. When TBPBP was simultaneously employed with SA powder, RPUFs could obtain better fire and smoke safety than adding TBPBP or SA powder alone due to the difficult flammability and the adsorption of flue gas of SA powder with a three–dimensional porous structure. A possible flame–retardant mechanism of RPUF–T45@SA20 is shown in Scheme 3.

## 4. Conclusions

TBPBP, as a phosphorus–nitrogen–containing reactive–type flame retardant, was successfully synthesized via the characterization of LC/MS, FT–IR, NMR and TGA. The introduction of TBPBP remarkably improved most of the physical and flame retardant properties via playing a flame–retardant performance in both condensed and gaseous phases. However, in terms of the instantaneous release of harmful gases, for example, CO, TBPBP had a striking weakness. To overcome these problems, the simultaneous utilization of TBPBP and SA powder (fabricated RPUF–T45@SA20) not only further enhanced the physical and flame–retardant properties, but also apparently reduced the instantaneous release of harmful gases compared to the one only using TBPBP. Meanwhile, the compressive strength of RPUF–T45@SA20 was increased to 457 kPa from 236 kPa (neat RPUF), and the LOI value was increased to 27.7 (RPUF–T45@SA20) from 19.2 of neat RPUF, and UL–94 testing of RPUF–T45@SA20 reached the V–0 rank. The total heat release (THR) and total smoke production (TSP) of RPUF–T45@SA20 were, respectively, reduced by 44.66% and 51.89% compared to those of neat RPUF. Thus, this study is significant for the application of modified castor oil–based rigid polyurethane foams incorporated with TBPBP and SA powder in the building insulation field.

## Figures and Tables

**Table 1:** The formulation of RPUFs.

Samples	MCOP	PEG–200	TBPBP	Deionized Water	DMP–30	Si–Oil	SA Powder	pMDI
neat RPUF	80	20	0	4	2	4	0	146.79
RPUF–T15	65	20	15	4	2	4	0	149.50
RPUF–T30	50	20	30	4	2	4	0	152.20
RPUF–T45	35	20	45	4	2	4	0	154.91
RPUF–SA20	80	20	0	4	2	4	20	146.79
RPUF–T45@SA20	35	20	45	4	2	4	20	154.91

1. The unit of all reagents is gram. 2. The abbreviation of the raw material corresponds to the full name in Section 2.1.

**Table 2:** Density, compressive strength and thermal conductivity of RPUFs.

Sample	Density (kg/m^3^)	Compressive Strength (kPa)	Thermal Conductivity Coefficient W/(mK)
Neat RPUF	36.7 ± 0.9	236 ± 29	31.67 ∗ 10^−3^
RPUF–T15	37.2 ± 0.5	317 ± 16	32.13 ∗ 10^−3^
RPUF–T30	37.9 ± 0.6	389 ± 31	32.79 ∗ 10^−3^
RPUF–T45	38.2 ± 0.7	422 ± 30	34.65 ∗ 10^−3^
RPUF–SA20	42.5 ± 1.2	396 ± 18	35.21 ∗ 10^−3^
RPUF–T45@SA20	43.9 ± 1.0	457 ± 24	36.95 ∗ 10^−3^

**Table 3:** TGA and DTG data of RPUFs under N_2_.

Samples	T_5%_	T_50%_	Rmax_1_/Tmax_1_	Rmax_2_/Tmax_2_	Residue (%)
	°C	°C	%∙min^−1^/°C	%∙min^−1^/°C	At 700 °C
neat RPUF	249.04	367.04	−7.03/307.04	−2.88/425.04	16.89
RPUF–T15	221.04	341.04	−5.91/303.04	−2.25/397.04	16.28
RPUF–T30	213.04	359.04	−5.07/311.04	−2.58/389.04	21.14
RPUF–T45	199.04	375.04	−3.87/307.04	−3.27/379.04	24.79
RPUF–SA20	261.04	401.04	−5.83/305.04	−2.92/425.04	24.53
RPUF–T45@SA20	207.04	385.04	−4.01/307.04	−2.62/383.04	30.90

**Table 4:** TGA and DTG data of RPUFs under air.

Samples	T_5%_	T_50%_	Rmax_1_/Tmax_1_	Rmax_2_/Tmax^2^	Residue (%)
	°C	°C	%∙min^−1^/°C	%∙min^−1^/°C	At 700 °C
neat RPUF	260.48	452.48	−7.29/294.48	−7.06/502.48	0.47%
RPUF–T15	218.48	446.48	−5.10/280.48	−5.51/506.48	2.09%
RPUF–T30	212.48	456.48	−4.50/278.48	−6.01/498.48	4.08%
RPUF–T45	194.48	450.48	−4.06/272.48	−6.21/480.48	6.49%
RPUF–SA20	254.48	440.48	−7.31/290.47	−6.34/492.48	3.48%
RPUF–T45@SA20	210.48	468.48	−3.48/274.48	−5.16/498.48	10.96%

**Table 5:** LOI values and UL–94 grades of RPUFs.

Sample	LOI(%)	UL–94
Neat RPUF	19.2	N.R.
RPUF–T15	22.9	V–0
RPUF–T30	23.4	V–0
RPUF–T45	25.6	V–0
RPUF–SA20	20.3	N.R.
RPUF–T45@SA20	27.7	V–0

Note: LOI values of RPUFs are average values of parallel tests.

**Table 6:** Characteristic CC data of neat RPUF, RPUF–T45, RPUF–SA20, and RPUF–T45@SA20.

Samples	TTI	THR	TSP	Av–EHC	Residue
	s	MJ/m^2^	M^Y+^	MJ/kg	%
neat RPUF	0	30.54	7.69	21.36	3.17
RPUF–T45	3	20.67	5.07	15.75	9.29
RPUF–SA20	0	26.05	5.78	19.69	4.03
RPUF–T45@SA20	4	16.90	3.71	13.04	11.76

## Data Availability

Not applicable.

## Supplementary Materials

The following are available online at https://www.mdpi.com/article/10.3390/polym13132140/s1, Scheme S1: The reaction equation for preparing RPUFs. Figure S1: EDXS images of RPUFs. Figure S2: EDXS images of the char residues of RPUFs. Table S1: The retention time and chemical structure of main pyrolysis products of RPUF–T45.
