# Supplementary material for: Enhancing the Fire Safety and Smoke Safety of Bio–Based Rigid Polyurethane Foam via Inserting a Reactive Flame Retardant Containing P@N and Blending Silica Aerogel Powder
Source: Polymers (Basel). 2021 Jun 29;13(13):2140. doi: 10.3390/polym13132140 (PMC8271911; doi:10.3390/polym13132140)
Supplement: Supplementary file 1 [file polymers-13-02140-s001.zip › polymers-1272907-supplementary.pdf]

Enhancing the fire safety and smoke safety of bio-based rigid polyurethane foam via inserting a reactive flame retardant containing P@N and blending silica aerogel powder

Guangxu Bo, Xiaoling Xu, Xiaoke Tian, Jiao Wu, Yunjun Yan*

Key Laboratory of Molecular Biophysics of the Ministry of Education, College of Life Science and Technology, Huazhong University of Science and Technology, Wuhan 430074, China.

* Corresponding author.

E-mail address: yanyunjun@hust.edu.cn(Y.J.)

Scheme S1. The reaction equation for preparing RPUFs.

Figure S1. EDXS images of RPUFs.

Figure S2. EDXS images of the char residues of RPUFs.

Table S1 The retention time and chemical structure of main pyrolysis products of RPUF-T45.

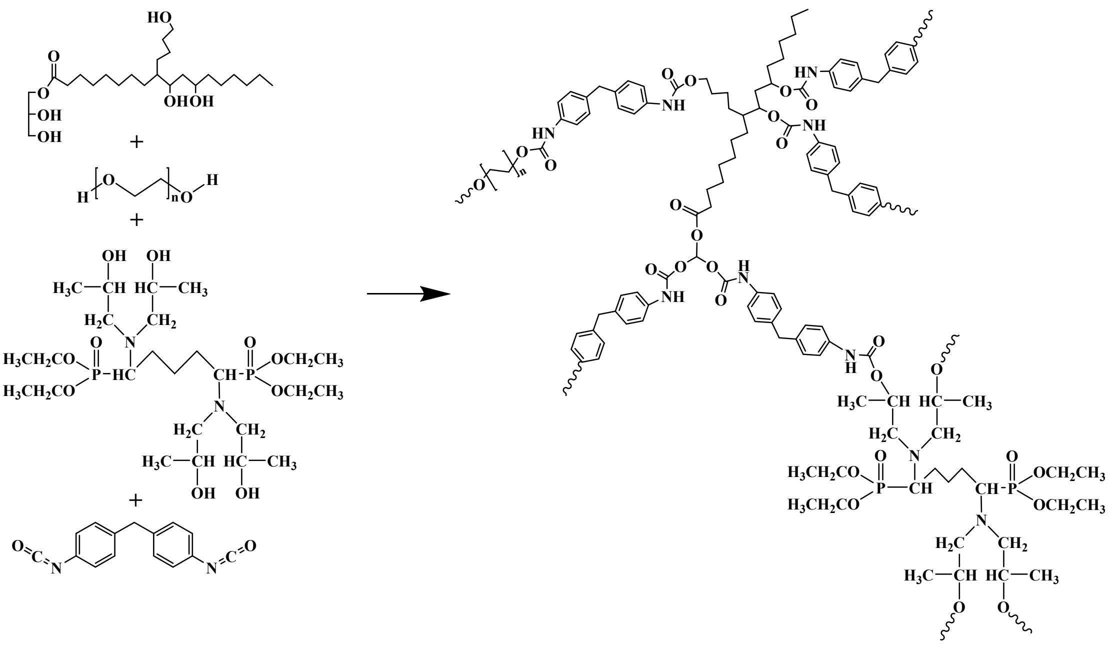

Scheme S1. The reaction equation for preparing RPUFs.

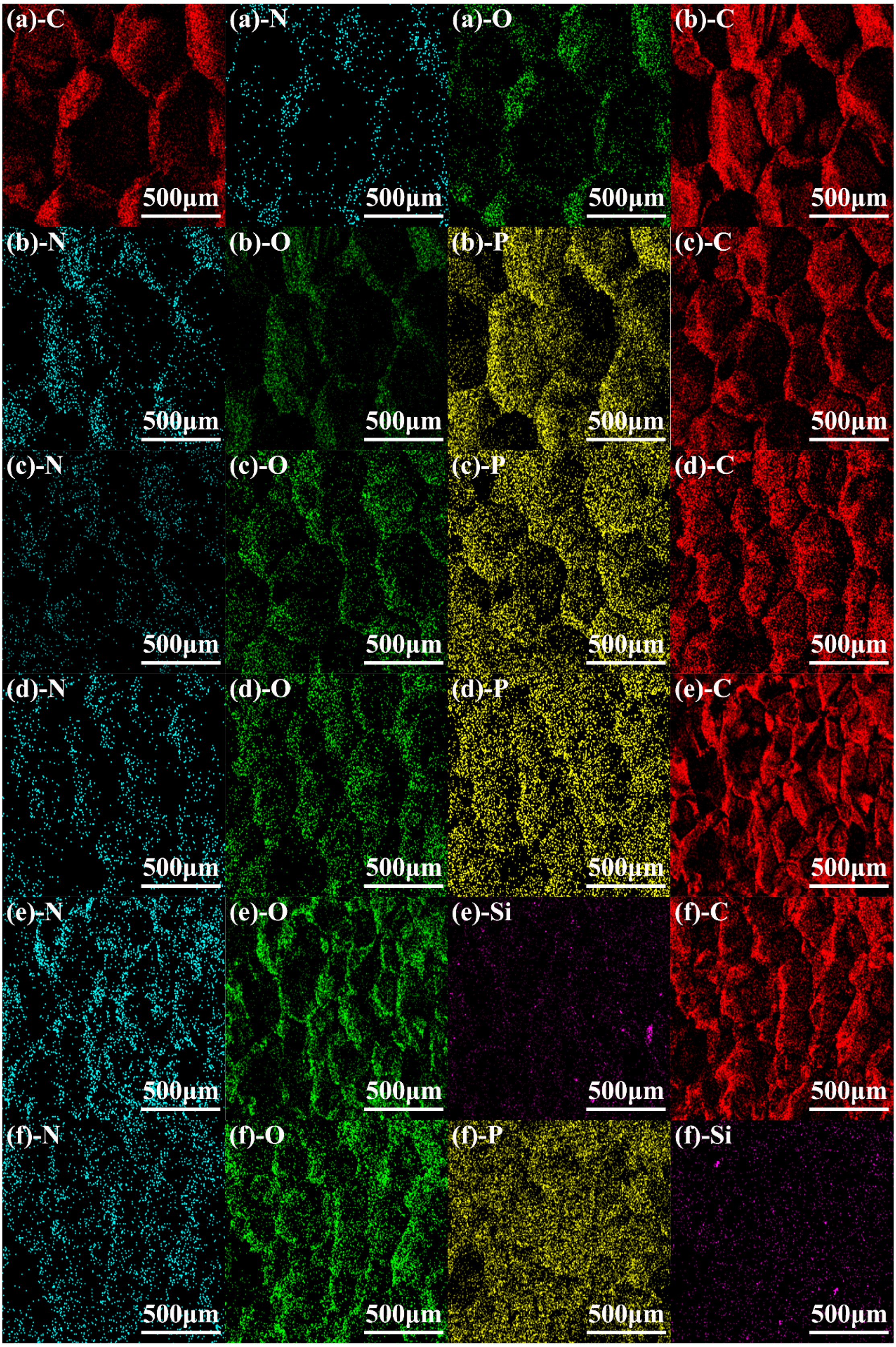

Figure S1. EDXS images of RPUFs

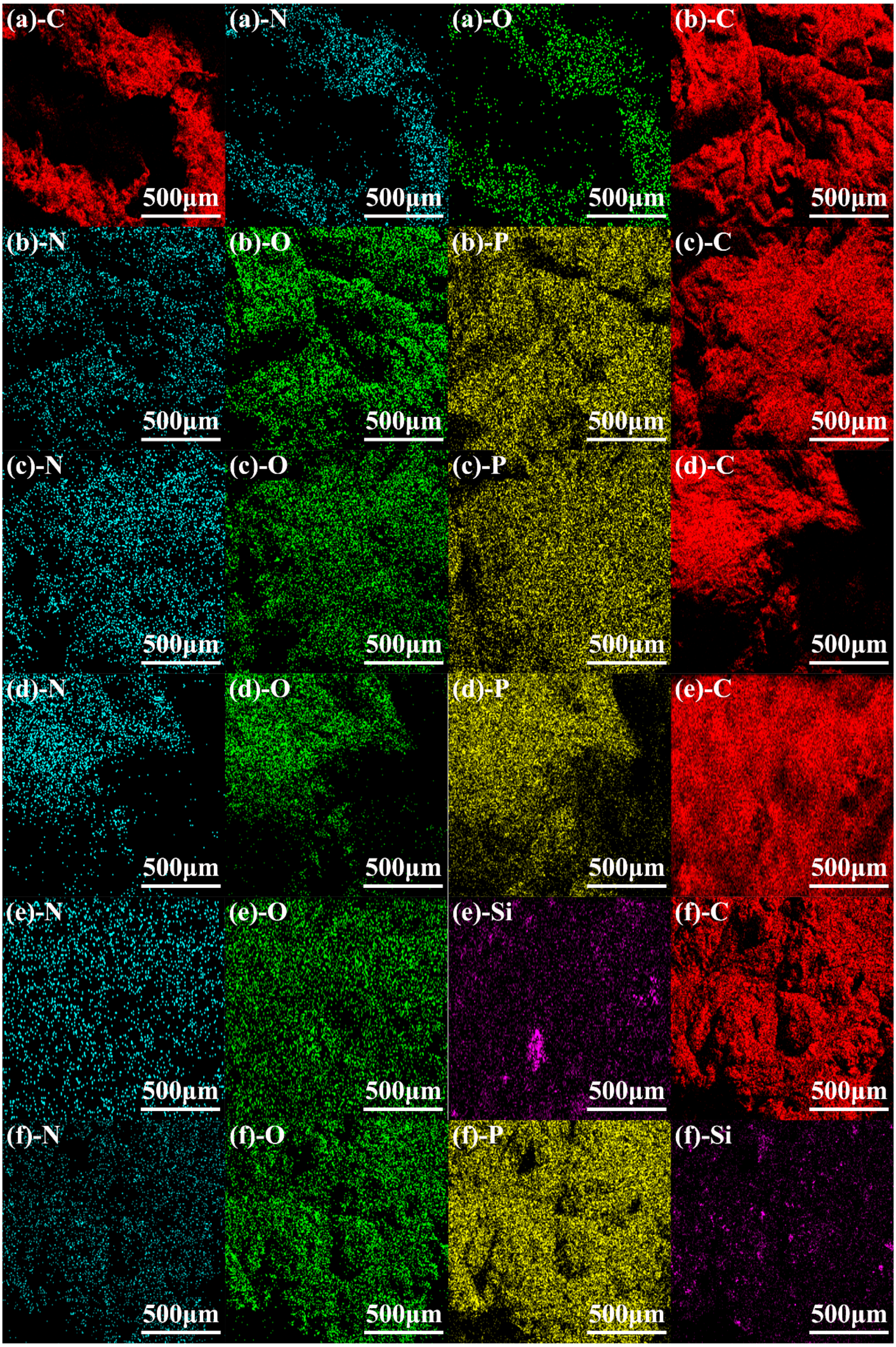

Figure S2. EDXS images of the char residues of RPUFs.

Table S1. The retention time and chemical structure of main pyrolysis products of RPUF-T45.

Peak	Retention Time (min)	Structure	Peak	Retention Time (min)	Structure
A	1.34	CO_2	H	11.07	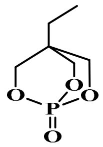
B	1.42	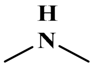	I	11.38	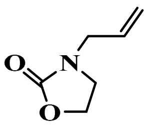
C	1.45	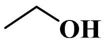	J	14.05	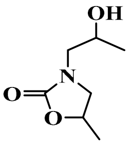
D	2.68	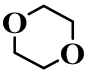	K	14.87	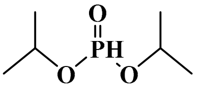
E	6.55	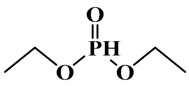	L	18.72	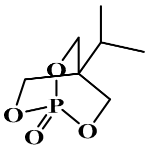
F	7.07	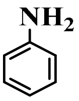	M	21.16	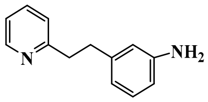
G	8.73	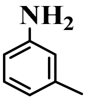	N	21.79	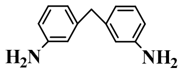
